# A pH-eQTL Interaction at the *RIT2*–*SYT4* Parkinson’s Disease Risk Locus in the Substantia Nigra

**DOI:** 10.3389/fnagi.2021.690632

**Published:** 2021-07-09

**Authors:** Sejal Patel, Derek Howard, Leon French

**Affiliations:** ^1^Krembil Centre for Neuroinformatics, Centre for Addiction and Mental Health, Toronto, ON, Canada; ^2^Campbell Family Mental Health Research Institute, Centre for Addiction and Mental Health, Toronto, ON, Canada; ^3^Department of Psychiatry, University of Toronto, Toronto, ON, Canada; ^4^Institute for Medical Science, University of Toronto, Toronto, ON, Canada

**Keywords:** *RIT2*, pH, rs12456492, substantia nigra (SN), Parkinson’s disease, *SYT4*, eQTL analysis, gene expression

## Abstract

Parkinson’s disease causes severe motor and cognitive disabilities that result from the progressive loss of dopamine neurons in the substantia nigra. The rs12456492 variant in the *RIT2* gene has been repeatedly associated with increased risk for Parkinson’s disease. From a transcriptomic perspective, a meta-analysis found that *RIT2* gene expression is correlated with pH in the human brain. To assess these pH associations in relation to Parkinson’s disease risk, we examined the two datasets that assayed rs12456492, gene expression, and pH in the postmortem human brain. Using the BrainEAC dataset, we replicate the positive correlation between *RIT2* gene expression and pH in the human brain (*n* = 100). Furthermore, we found that the relationship between expression and pH is influenced by rs12456492. When tested across ten brain regions, this interaction is specifically found in the substantia nigra. A similar association was found for the co-localized *SYT4* gene. In addition, *SYT4* associations are stronger in a combined model with both genes, and the *SYT4* interaction appears to be specific to males. In the Genotype-Tissue Expression (GTEx) dataset, the pH associations involving rs12456492 and expression of either *SYT4* and *RIT2* were not seen. This null finding may be due to the short postmortem intervals of the GTEx tissue samples. In the BrainEAC data, we tested the effect of postmortem interval and only observed the interactions in samples with the longer intervals. These previously unknown associations suggest novel roles for rs12456492, *RIT2*, and *SYT4* in the regulation and response to pH in the substantia nigra.

## Introduction

Parkinson’s disease (PD) is a common neurodegenerative disease characterized by the loss of dopamine neurons in the substantia nigra. Individuals with PD show severe motor and cognitive disabilities. The etiology of PD is complex, with multiple genetic (Nalls et al., 2019) and environmental risk factors [reviewed in Bellou et al. (2016)]. A deeper understanding of interactions between these factors may reveal new insights into PD pathophysiology.

**FIGURE 1:**
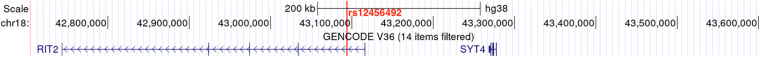
Genomic context of rs12456492 obtained from the homo sapiens genome assembly hg18 and visualized with the UCSC Genome Browser (Kent et al., 2002). The red vertical line marks the position of rs12456492.

Recent genome-wide association studies (GWAS) of sporadic PD found that common genetic variants explain 16–36% of heritable risk (Nalls et al., 2019). Most experimental studies have focused on genes that have been associated with both monogenic and sporadic forms of PD. These include alpha-synuclein (*SNCA*), leucine-rich repeat kinase 2 (*LRRK2*), and glucosylceramidase beta (*GBA*) (Chang et al., 2017). However, over 90 independent risk signals have been identified. These recent GWAS hits are underexplored in the context of PD. For example, the rs12456492 polymorphism was first associated with PD in a 2011 GWAS study (Do et al., 2011). Subsequent studies have replicated this locus on chromosome 18, confirming an association with PD (Nalls et al., 2019). The most common allele, A, with a frequency of 68%, is associated with a lower incidence of PD (odds ratio: 0.906). Conversely, the minor G allele is associated with PD risk. As shown in Figure 1, rs12456492 is located within an intron of the Ras Like Without CAAX 2 (*RIT2*) gene and is in linkage disequilibrium with polymorphisms in the nearby Synaptotagmin 4 gene (*SYT4*) (Pankratz et al., 2012). *RIT2* binds to dopamine transporters, guanosine triphosphate, and calmodulin (Lee et al., 1996; Navaroli et al., 2011). The *SYT4* gene is a member of the synaptotagmin family and regulates synaptic transmission (Dean et al., 2009). In the context of PD, Mendez et al. (2011) demonstrated that somatodendritic dopamine release depends on *SYT4*. However, the *RIT2* and *SYT4* genes have not been extensively characterized in relation to PD.

In a cross laboratory comparison of expression profiling data from normal human postmortem brains, Mistry and Pavlidis (2010) identified a robust correlation between *RIT2* and tissue pH. This meta-analysis included 11 studies that provided 421 cortical transcriptomes. Genome-wide, *RIT2* was ranked tenth on the pH up-regulation list of 15,845 genes. The regulation of pH within the brain is crucial for proper physiological functioning. Specifically, impairment in this regulation can alter neuronal state leading to physiopathological conditions [reviewed in Sinning and Hübner (2013)]. Thus, the Mistry and Pavlidis meta-analysis results suggest that *RIT2* may be involved in neural pH regulation and response.

Several studies have examined pH-dependent interactions in the context of Parkinson’s disease. For example, in comparison to neutral or physiological pH of 7 or 7.4, α-synuclein aggregation and stability are increased in acidic conditions (pH 4–5) (Pham et al., 2009; Lv et al., 2016). In addition, β-synuclein, an inhibitor of α-synuclein aggregation, is sensitive to pH and forms fibrils in mildly acidic pH (5.8) (Moriarty et al., 2017; Williams et al., 2018). Using quantum chemical methods, Umek et al. (2018) determined that an acidic environment is required to prevent dopamine autoxidation. Kinetic modeling has also revealed that pH interactions with iron and dopamine could lead to oxidative stress (Sun et al., 2018). Caffeine consumption is associated with mild alkalosis (Tajima, 2010) and a decreased risk of PD [reviewed in Costa et al. (2010)]. We also note that *RIT2* is differentially co-expressed with interferon-gamma signaling genes in substantia nigra samples from PD cases (Liscovitch and French, 2014). While indirect, interferon-gamma is acid-labile, and its overexpression in mice causes nigrostriatal neurodegeneration (Piasecki, 1999; Chakrabarty et al., 2011). Mitochondria, which internally maintain an alkaline pH, are thought to be dysfunctional in PD [reviewed in Chen et al. (2019)]. Mitochondrial dysfunction can lead to oxidative stress, resulting in lactic and intraneuronal acidosis (Koga et al., 2006; Arias et al., 2008; Balut et al., 2008). Recently, Rango et al. found that carriers of *PINK1* mutations, which are associated with early-onset PD, have abnormal pH levels in the visual cortex. Specifically, carriers of homozygous *PINK1* mutations had a higher pH at rest when compared to healthy controls and patients without *PINK1* mutations. Unlike healthy controls, pH did not increase upon activation in homozygous *PINK1* mutation carriers (Rango et al., 2020). *PARK7* (*DJ-1*), another early-onset PD gene, is associated with pH. Specifically, acidic isoforms appear after oxidative stress (Bandopadhyay et al., 2004; Canet-Avilés et al., 2004). Taken together, these findings motivate a deeper characterization of pH, *SYT4*, and *RIT2* in the context of PD.

**FIGURE 2:**
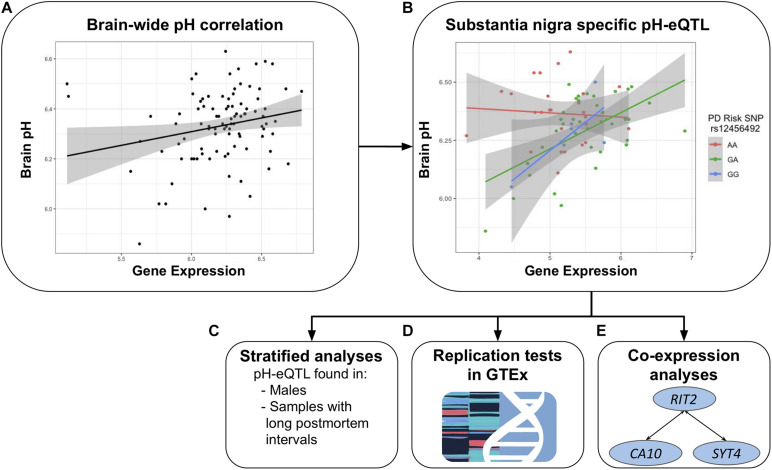
Overview of the study. Focused on *RIT2* and *SYT4*, the relationship between pH and gene expression was tested brain-wide **(A)**. Region-specific tests revealed a pH-eQTL involving the PD risk polymorphism rs12456492 in the substantia nigra **(B)**. When the sample is stratified, this relationship is only observed within males and samples with longer post mortem intervals **(C)**. GTEx was used for replication tests **(D)**. Lastly, we performed a further analysis that identified co-expression between *RIT2, SYT4*, and *CA10* **(E)**.

In this study, we use the BrainEAC data to replicate the correlation between *RIT2* expression and pH. In addition, we further test for associations between *SYT4* and pH. We characterize interactions involving a co-localized genetic risk variant for PD, pH, *RIT2*, and *SYT4* gene expression. We explore these interactions in two independent postmortem brain datasets and test the impact of PMI and sex. We also perform co-expression searches to associate genes of known function to *RIT2* and *SYT4*. Figure 2 provides an overview of these analyses.

## Materials and Methods

### BrainEAC Dataset

Phenotype, genome-wide expression, and genotype information were obtained from The Brain expression quantitative trait loci (eQTL) Almanac (BrainEAC) project. This data from the United Kingdom Brain Expression Consortium was generated to investigate genetic regulation and alternative splicing. The consortium assayed genome-wide expression in ten brain regions using Affymetrix Exon 1.0 ST arrays (Illumina, San Diego, CA, United States) from 134 neuropathologically normal donors (Ramasamy et al., 2014). We extracted genotype data and expression values for the *RIT2*, *SYT4*, and *CA10* genes from the BrainEAC web-based resource^1^. Age, sex, postmortem interval (PMI), RNA integrity number (RIN), cause of death, and pH data were obtained from Gene Expression Omnibus (GSE46706) (Edgar et al., 2002). Of the 134 brains, we restricted our analyses to the 100 brains from the Medical Research Council Sudden Death Brain and Tissue Bank in Edinburgh, United Kingdom (Millar et al., 2007) with pH and genotype data. For each brain, pH was measured in the lateral ventricle because it is known to be stable across brain regions (Trabzuni et al., 2012). This study measured pH with a Hanna HI8424 portable pH meter with a glass bodied electrode (Fisher Scientific, Loughborough, United Kingdom) (Trabzuni et al., 2012). All ten regions were not sampled in all the brains, resulting in 73 substantia nigra samples. The median age of death is 55 years (interquartile range: 44–61), with 76.7% composed of males. Demographic, genotype and other information for this sample are shown in Table 1.

**TABLE 1:** Demographic and genotype statistics for the BrainEAC and genotype-tissue expression (GTEx) datasets.

	BrainEAC	GTEx
N	73	113
Females (%)	17 (23.3)	33 (29.2)
Age ± SD	51.2 ± 15.2	57.9 ± 10.9
RIN ± SD	3.87 ± 1.18	6.62 ± 0.73
PMI ± SD	56.29 + 17.7	14.09 ± 4.21
pH ± SD	6.28 ± 0.22	6.2 ± 0.25
**rs12456492 genotype (%)**		
AA	28 (38.4)	51 (45.1)
GA	39 (53.4)	52 (46.0)
GG	6 (8.2)	10 (8.8)

### GTEx Dataset

The Genotype-Tissue Expression (GTEx^2^) project was designed to identify eQTL’s in diverse human tissues (Lonsdale et al., 2013). We extracted version 8 of GTEx data, which included expression data for the substantia nigra and genotype data for PD risk single nucleotide polymorphism (SNP) rs12456492, resulting in a sample size of 113. Tissue samples were collected from non-diseased postmortem brain samples. RNA sequencing expression data was obtained from the GTEx Portal and log normalized (filename: GTEx_Analysis_2017-06-05_v8_RNASeQCv1.1.9_gene_tpm.gct). Genotype information was obtained from whole-exome sequencing by the GTEx consortium (filename: GTEx_Analysis_2017-06-05_v8_WholeExomeSeq_979Indiv_VEP_annot.vcf). Additional information extracted from the GTEx Portal included age, sex, PMI, RIN, and pH (measured in the cerebellum) (filename: GTEx_Analysis_v8_Annotations_SampleAttributesDS.txt). The median age of death is 60 years (interquartile range: 53–66), with 70.8% composed of males. Additional statistics for this dataset are shown in Table 1.

### Co-expression Meta-Analysis Tools

Search-Based Exploration of Expression Compendium for Humans (SEEK^3^) was used to identify top genes co-expressed with *RIT2*. The top-ranked dataset from the co-expression result, GSE20146, was downloaded to characterize correlations between *RIT2*, *SYT4*, and *CA10*. Normalized expression data for GSE20146 was obtained from the Gemma bioinformatics system^4^, using the filter option, which resulted in the removal of one outlier (GSM505262) (Lim et al., 2021).

### Statistical Analysis

Pearson correlation and ordinary least square linear models were performed in R Core (2013). The R software environment for statistical computing and graphics was also used for plotting^5^.

## Results

### Brain-Wide *RIT2* and *SYT4* Gene Expression Is Correlated With pH

**FIGURE 3:**
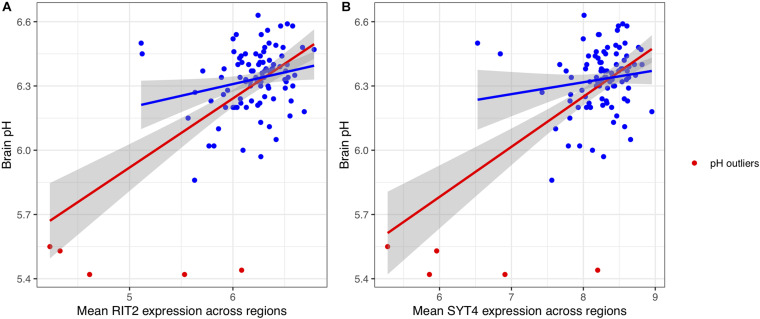
Scatter plots of the relationship between pH with *RIT2* **(A)** and *SYT4* **(B)** gene expression. pH outliers are colored red. Lines representing linear fits with (red) and without outliers (blue) include shaded areas marking the 0.95 confidence intervals.

To attempt replication of the association between pH and *RIT2* and additionally test *SYT4*, we examined their relationships. The gene expression of *RIT2* and *SYT4* was averaged across all ten brain regions assayed in the BrainEAC study (*n* = 100 brains, sample information provided in Table 1). We observed a broad range of pH values (5.42–6.63, measured in the lateral ventricle). The most common cause of death is ischemic heart disease (*n* = 60), and cause of death was not associated with brain pH (ANOVA, *p* = 0.48). Gene expression and brain pH was correlated for *RIT2* (*r* = 0.59, *p* < 0.0001), and *SYT4* (*r* = 0.58, *p* < 0.0001). As seen in Figure 3, five brains had abnormally low pH values, with pH values were lower than two standard deviations from the mean (ph < 5.8). To prevent these outliers from skewing our downstream results, we have removed them from all subsequent analyses. After removal of these pH outlier brains, *RIT2* remains correlated with pH (*r* = 0.22, *p* < 0.04), however, *SYT4* was no longer correlated with brain pH (*SYT4*: *r* = 0.14, *p* = 0.17). Thus, in agreement with Mistry and Pavlidis, we also observe a correlation between postmortem brain pH and *RIT2* gene expression.

Although pH was measured at the lateral ventricle, it is known that pH is relatively consistent brain-wide (Trabzuni et al., 2012). However, we tested if the correlation with gene expression varies across the ten brain regions that were transcriptomically profiled. For each individual brain region, *RIT2* was most correlated with pH in the thalamus (*n* = 90, *r* = 0.29, *p* = 0.005, *p*_*FDR*_ = 0.055) and substantia nigra (*n* = 70, *r* = 0.27, *p* = 0.026, *p*_*FDR*_ = 0.13). In contrast, the correlation between *SYT4* expression and pH was not statistically significant in any of the 10 regions. *SYT4* correlation was high but not significant in the thalamus (*r* = 0.20, *p* = 0.057). White matter and regions enriched for white matter (putamen and medulla) had the lowest pH correlations for *RIT2* gene expression, whereas the medulla and substantia nigra had the lowest pH correlation for *SYT4*.

### rs12456492 Influences the Association Between *RIT2* and *SYT4* Expression and pH in the Substantia Nigra

**FIGURE 4:**
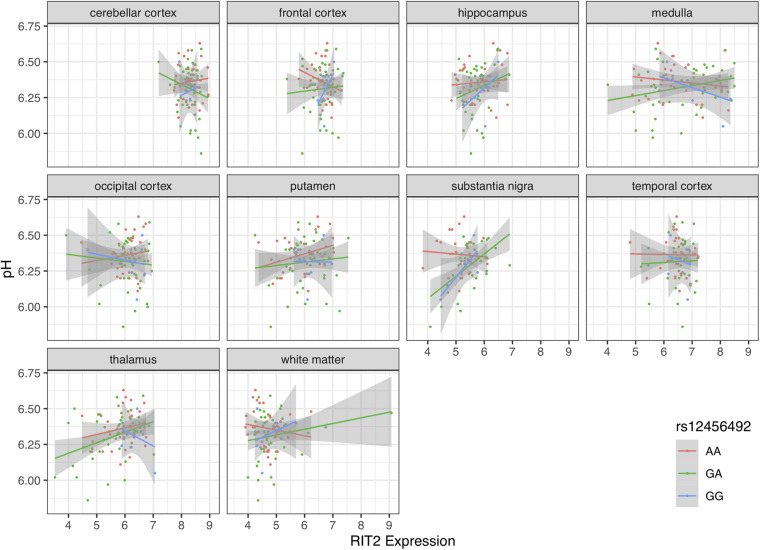
Scatter plot of pH and *RIT2* gene expression based on rs12456492 genotype for 10 brain regions. Genotype groups are colored with lines representing linear fits with shaded areas marking the 0.95 confidence intervals.

**FIGURE 5:**
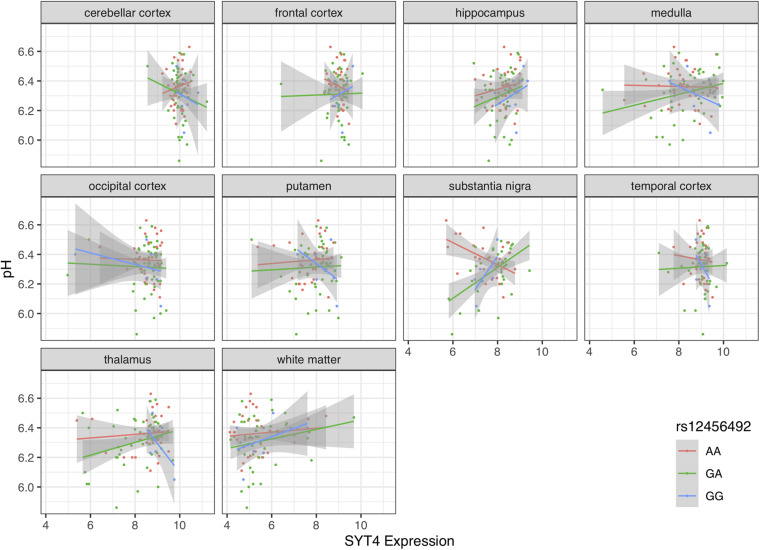
Scatter plot of pH and *SYT4* gene expression based on rs12456492 genotype for 10 brain regions. Genotype groups are colored with lines representing linear fits with shaded areas marking the 0.95 confidence intervals.

Motivated by the correlations between brain pH, *RIT2*, and *SYT4*, we next tested for associations with the co-located PD risk variant. Genotype at rs12456492 by itself was associated with brain pH (*B* = 0.06, *p* = 0.043). We next tested if this neighboring PD risk variant influenced the correlations between gene expression and pH. As depicted in Figures 4, 5, an interaction between pH, rs12456492 and either *RIT2* or *SYT4* expression, was observed in the substantia nigra (*RIT2*: *B* = −0.15, *p* < 0.007, *p*_FDR_ < 0.07, *SYT4*: *B* = −0.16, *p* < 0.0001, *p*_*FDR*_ < 0.001) but none of the other profiled regions. As shown in Table 2, after accounting for the covariates of sex, age, PMI and RIN, these signals remain. Compared to all other terms in the model, the pH-eQTL interaction for either *RIT2* or *SYT4* expression is the most significant (*p* < 0.005) (Table 2). Specifically, in the BrainEAC data, individuals carrying the risk allele (AG and GG) had a positive correlation between gene expression and pH, but non-carriers did not. This interaction suggests a weaker coupling between pH, *RIT2*, and *SYT4* expression in the substantia nigra may be protective.

**TABLE 2:** Effects of Rs12456492, *RIT2*, and *SYT4* gene expression on brain pH, with sex, age, postmortem interval (PMI) and RNA integrity number (RIN) as covariates.

	Model 1	Model 2	Model 3	Model 4	Model 5	Model 6
Rs12456492 (SNP)	0.06	0.94	1.38	0.90	1.23	1.23
	(0.006–0.12),	(0.35–1.53),	(0.84–1.92),	(0.31–1.49),	(0.71–1.77),	(0.64–1.81),
	*p* < 0.04	*p* < 0.003	*p* < 0.00001	*p* < 0.004	*p* < 0.00002	*p* < 0.0001
RIT2		0.30		0.34	0.11	0.10
		(0.14–0.45),		(0.18–0.51),	(0.02–0.20),	(−0.12 to 0.32),
		*p* < 0.0004		*p* < 0.0001	*p* < 0.03	*p* < 0.37
SYT4			0.26	−0.05	0.17	0.18
			(0.16–0.37),	(−0.12 to 0.01),	(0.05–0.30),	(0.02–0.34),
			*p* < 0.00001	*p* = 0.11	*p* < 0.006	*p* < 0.03
RIT2 × SNP		−0.17		−0.16		0.01
		(−0.23 to −0.05),		(−0.27 to −0.04),		(−0.14 to 0.15),
		*p* < 0.005		*p* < 0.007		*p* = 0.93
SYT4 × SNP			−0.17		−0.15	−0.16
			(−0.24 to −0.10),		(−0.22 to −0.08),	(−0.26 to −0.06),
			*p* < 0.00001		*p* < 0.00006	*p* < 0.003
Adjusted *R*^2^	0.013	0.20	0.28	0.22	0.33	0.32

To investigate the combined influences of *RIT2* and *SYT4* on pH level, we tested three additional models (Table 2, models 4–6). After including covariates, the addition of *SYT4* gene expression resulted in a slightly better fit for the *RIT2* interaction model (Model 2 vs 4, *R*^2^ from 0.2 to 0.22, *p* = 0.11). Similarly, the addition of *RIT2* expression explained slightly more variance in the *SYT4* interaction model (Model 3 vs 5, *R*^2^ from 0.28 to 0.33, *p* = 0.02). Models that have the *SYT4* genetic interaction explain more variance than those that include the *RIT2* interaction. Furthermore, in a model with all tested terms, the *RIT2* genetic interaction term is no longer significant (Model 6).

### Sex-Specific Signals

**FIGURE 6:**
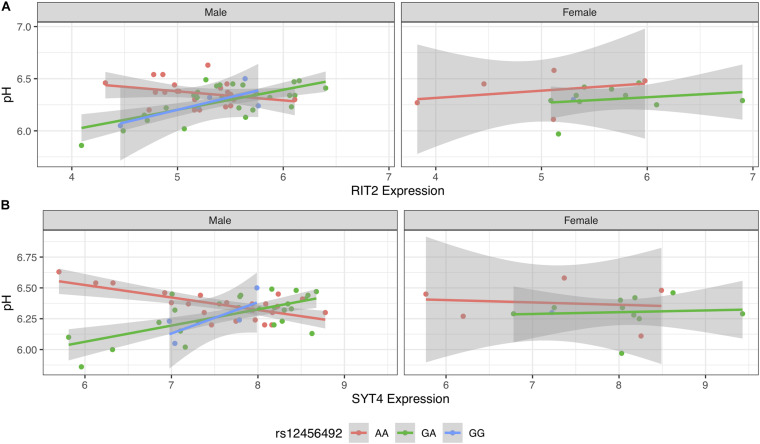
Scatter plot of pH and gene expression grouped by risk SNP genotype and stratified by sex within BrainEAC sample for *RIT2* **(A)** and *SYT4* **(B)**. Genotype groups are colored with lines representing linear fits with shaded areas marking the 0.95 confidence intervals.

To investigate if sex influenced the pH-eQTL interaction, we tested a three-way interaction between gene expression, genotype, and gender. This added interaction term was not significant for *RIT2* [*B* = 0.21 (−0.05 to 0.48), *p* = 0.11], but was for *SYT4* [*B* = 0.18 (0.006–0.35), *p* < 0.042] when added to Models 2 and 3. We additionally stratified our analyses to test for sex-specific effects on Models 2 and 3 (Figure 6). This yielded 54 male samples and a small group of 16 female samples that lack individuals carrying the GG genotype. In both models, pH-eQTL interactions were significant for males (*RIT2*: *B* = −0.21, *p* < 0.0001, Figure 6A; *SYT4*: *B* = −0.21, *p* < 0.003, Figure 6B) but not females (*RIT2*: *B* = −0.03, *p* = 0.88, Figure 6A; *SYT4*: *B* = −0.09, *p* = 0.47, Figure 6B). Overall, the *SYT4* pH-eQTL appears to be sex-specific, but this analysis is limited by sample size.

### rs12456492 Is Not Associated With *RIT2* and *SYT4* Expression and pH Within the Substantia Nigra in the GTEx Dataset

Next, we used the GTEx dataset to test for replication of our findings from the BrainEAC sample. We obtained gene expression data for *RIT2* and *SYT4* in the substantia nigra region, along with genotype data for the PD risk SNP rs12456492 yielding a sample size of 113. The pH ranged from 5.58 to 6.79, with a mean of 6.20. Similarly, we removed pH outliers from the samples using the same criteria used for the BrainEAC dataset, resulting in 104 samples. Although the location of pH measurement (cerebellum) and method of gene expression profiling (RNA sequencing) varies, we tested the same models in this dataset.

In the substantia nigra, the correlation between gene expression of *RIT2* or *SYT4* with pH was significant (*RIT2*: *r* = 0.27, *p* < 0.006; *SYT4*: *r* = 0.31, *p* < 0.002). Next, we applied linear models on the GTEx data to first examine the influence of the risk SNP on pH level. The risk SNP by itself was not a significant predictor of pH (*p* = 0.14). Furthermore interaction between pH, rs12456492 and either *RIT2* or *STY4* was not observed (*RIT2*: *t*-stat = −1.10, *p* = 0.28, *SYT4*: *t*-stat = −0.84, *p* = 0.40). After accounting for the covariates (sex, age, RIN, and PMI), the gene expression and SNP interactions were still not statistically significant terms in the models. Also, sex-specific signals were not observed in the GTEx substantia nigra samples.

### Shorter PMI Values in GTEx

**FIGURE 7:**
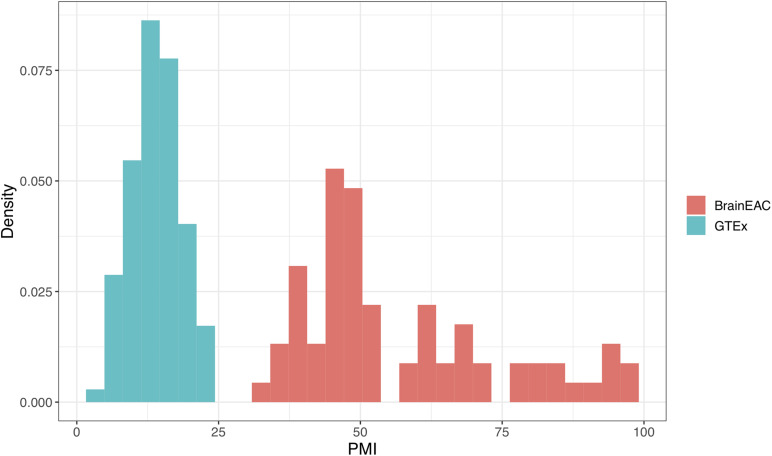
Density plot of postmortem interval (PMI) values in hours. The genotype-tissue expression (GTEx) and BrainEAC samples are colored in blue and red, respectively.

To explain the failed replication in the GTEx dataset, we examined the differences between the two cohorts. We noticed that PMI was longer in BrainEAC when compared to the GTEx samples. For BrainEAC, PMI ranged from 31 to 99 h, whereas GTEx ranged from 4.78 to 23.13 h. Hence there was no overlap between the two datasets, as seen in Figure 7.

### PMI Influences pH-eQTL Strength

**FIGURE 8:**
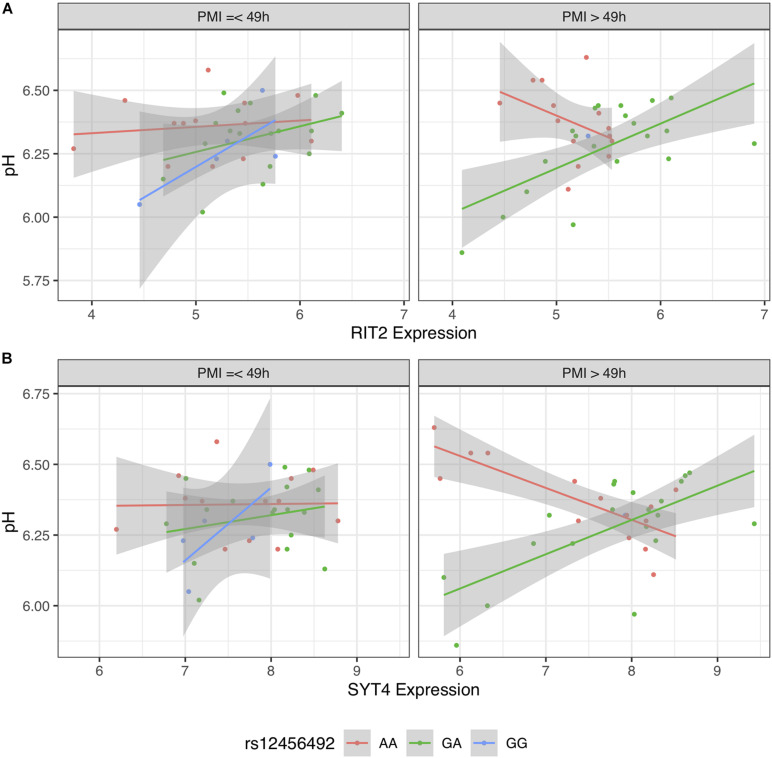
Scatter plot of pH and gene expression grouped by risk single nucleotide polymorphism (SNP) genotype and stratified PMI within BrainEAC sample for *RIT2* **(A)** and *SYT4* **(B)**. Genotype groups are colored with lines representing linear fits with shaded areas marking the 0.95 confidence interval.

To further investigate this difference, we stratified BrainEAC based on PMI. We split the sample based on the median PMI (49 h). Using Models 2 and 3, interactions between gene expression and genotype were not statistically significant in the short PMI group (*RIT2*: *p* = 0.15; *SYT4*: *p* = 0.24) but were in the long PMI group (*p* < 0.01 for both genes). The interaction between SNP, gene expression and PMI was not statistically significant for *RIT2* [*B* = −0.005 (−0.012 to 0.003), *p* = 0.20, Figure 8A], but was for *SYT4* [*B* = −0.004 (−0.008 to −0.0007), *p* < 0.02, Figure 8B]. In Figure 8, G allele carriers in the short and long PMI groups show a positive correlation between gene expression and pH level. Although the long PMI group lacks GG carriers, a switch from positive to negative correlation is observed when comparing the GA and AA groups. This suggests the PMI difference between the datasets may explain why the pH-eQTL interaction was not observed in the GTEx sample.

### CA10 Is Co-expressed With *SYT4* and *RIT2*

Using a co-expression search tool, the two genes most co-expressed with *RIT2* were carbonic anhydrase 10 (*CA10*) and *SYT4*. Of the 97 brain datasets that excluded cancer studies, based on co-expression, the most relevant dataset was a study of Parkinson’s disease [GSE20146, (Zheng et al., 2010)]. In this top dataset that assayed expression in the globus pallidus interna (*n* = 19), *RIT2* expression was correlated with *CA10* (*r* = 0.52, *p* < 0.03) and *SYT4* (*r* = 0.75, *p* < 0.0003), but *SYT4* was not co-expressed with *CA10* (*r* = 0.43, *p* = 0.065). Comparison of the correlation coefficients obtained from the PD cases and controls separately did not suggest differential co-expression. We note that *CA10* gene expression is not a significant predictor for pH when accounting for the other covariates (sex, age, PMI, and RIN) in either BrainEAC or GTEx substantia nigra samples. Therefore the addition of *CA10* does not improve the models in predicting pH.

## Discussion

This study replicates the positive correlation between *RIT2* gene expression and pH in the human brain. Furthermore, we found that a co-localized PD-associated genetic variant altered this relationship between expression and pH. When tested across ten brain regions, this interaction is specifically found in the substantia nigra, the primary location of PD pathology. A similar association was found for the neighboring *SYT4* gene. For non-carriers of the risk allele, the brain-wide positive correlation between gene expression and pH is inverted in the substantia nigra. In a combined model with both genes, the *SYT4* relationship is stronger. We attempted to replicate these findings using the GTEx dataset. However, the association between the risk allele and gene expression of either *SYT4* and *RIT2* with pH was not seen. We observed that the PMI values were longer in the BrainEAC cohort compared to the GTEx cohort. After stratifying the BrainEAC samples by PMI, we only observed the relationship in the longer PMI group. These associations implicate interactions between rs12456492, PMI, *RIT2*, and *SYT4* in regulating pH in the brain.

Known molecular mechanisms that could explain the observed pH-eQTL are lacking. One exception is a recent study of interactions between *RIT2* and *LRRK2* (Obergasteiger et al., 2021). Using postmortem gene expression data, Obergasteiger et al. observed down-regulation of *RIT2* in the substantia nigra of PD cases. In support, they observed reduced *RIT2* expression in neuroblastoma cell lines overexpressing *LRRK2*. Overexpression of *RIT2* and a PD-associated *LRRK2* mutant in these cells resulted in a higher number of autophagosomes and lysosomes. Both of these cellular components are acidic, suggesting autophagy-related regulation may underlie the pH interactions we observe.

Both *SYT4* and *CA10* are co-expressed with *RIT2*. Unlike *RIT2* and *SYT4*, carbonic anhydrases are known to regulate intracellular and extracellular pH (Wingo et al., 2001). However, *CA10* is catalytically inactive and forms complexes with synaptic proteins (Sjöblom et al., 1996; Nishimori et al., 2013; Sterky et al., 2017). In agreement, *CA10* gene expression was not a predictor for brain pH when added to either the *RIT2* or *SYT4* models. Recently, Payan-Gomez et al. performed a co-expression network analysis of the human prefrontal cortex. In this analysis that used brain samples from old and young individuals, *CA10* was identified as a central gene in the network (Payán-Gómez et al., 2019). A related gene, *CA2*, was found to be elevated in mitochondria from middle-aged mouse brain samples (Pollard et al., 2016). Furthermore, carbonic anhydrase inhibitors have been found to prevent amyloid beta-induced mitochondrial toxicity (Fossati et al., 2016; Solesio et al., 2018). Further study of interactions between *RIT2* and *CA10* may reveal pH regulation mechanisms relevant to PD.

When stratified by sex, the pH-eQTL relationship was not observed in females. This was more evident for *SYT4* than *RIT2*. The prevalence of PD is higher in males than females (Marras et al., 2018). Although our analysis was limited by sample size, we postulate that this sex-specific effect may help understand differences in PD incidence.

We suspect that we did not validate our findings in the GTEx dataset because of short PMI values in comparison to the BrainEAC samples. The differences between the datasets are apparent, as there is no overlap between the PMI values. When the BrainEAC cohort is split into short and long PMI groups, the results replicate the cohort differences. Specifically, the pH-eQTL relationship is observed in the samples with long but not short PMI. In agreement, studies of postmortem expression have found gene, and genotype-dependent associations with PMI (Zhu et al., 2017; Scott et al., 2020). Although we performed our analyses on neuropathologically normal brains, we speculate that a longer PMI value represents a neurodegenerative state that may mimic the nigral degeneration observed in the Parkinsonian brain. However, the neurodegenerative state caused by Parkinson’s disease is likely very different from the postmortem environment. Follow-up studies of postmortem samples or cell cultures derived from PD patients are warranted to test our findings in an experimental setting.

This study described a relationship between gene expression and pH that interacted with genetic variation. Our analysis of this pH-eQTL relationship is constrained to the *RIT2* locus that is associated with PD risk and is only observed in substantia nigra. Additional interactions with sex and PMI were observed for *SYT4* and, to a lesser degree, *RIT2*. These previously unknown associations suggest pH-associated roles for rs12456492, *RIT2*, and *SYT4* in the Parkinsonian brain.

## Data Availability Statement

Scripts and data for reproducing the majority of the analyses are publicly available online at https://github.com/Sejal24/PD_Manuscript_RIT2_SYT4_pH. The GTEx data is available via DBGap^6^ (Accession: phs000424.v8.p2).

## Author Contributions

SP and LF conceptualized the design of the study and were involved in data analysis and writing of the manuscript. SP, LF, and DH contributed to the data acquisition and editing of the manuscript. All authors contributed to the article and approved the submitted version.

## Conflict of Interest

LF owns shares in and has received consulting fees from Cortexyme Inc., a company that is developing gingipain inhibitors to treat neurodegenerative diseases. The remaining authors declare that the research was conducted in the absence of any commercial or financial relationships that could be construed as a potential conflict of interest.

## Footnotes

1 http://www.braineac.org/

2 https://gtexportal.org/

3 https://seek.princeton.edu/

4 https://gemma.msl.ubc.ca/

5 https://www.r-project.org/

6 https://www.ncbi.nlm.nih.gov/gap/

## References

[B1] Arias R. L. Sung M.-L. A. Vasylyev D. Zhang M.-Y. Albinson K. Kubek K. (2008). Amiloride is neuroprotective in an MPTP model of Parkinson’s disease. *Neurobiol. Dis.* 31 334–341. 10.1016/j.nbd.2008.05.008 18606547

[B2] Balut C. vandeVen M. Despa S. Lambrichts I. Ameloot M. Steels P. (2008). Measurement of cytosolic and mitochondrial pH in living cells during reversible metabolic inhibition. *Kidney Int.* 73 226–232. 10.1038/sj.ki.5002632 17978815

[B3] Bandopadhyay R. Kingsbury A. E. Cookson M. R. Reid A. R. Evans I. M. Hope A. D. (2004). The expression of DJ-1 (PARK7) in normal human CNS and idiopathic Parkinson’s disease. *Brain* 127 420–430. 10.1093/brain/awh054 14662519

[B4] Bellou V. Belbasis L. Tzoulaki I. Evangelou E. Ioannidis J. P. A. (2016). Environmental risk factors and Parkinson’s disease: an umbrella review of meta-analyses. *Parkinsonism Relat. Disord.* 23 1–9. 10.1016/j.parkreldis.2015.12.008 26739246

[B5] Canet-Avilés R. M. Wilson M. A. Miller D. W. Ahmad R. McLendon C. Bandyopadhyay S. (2004). The Parkinson’s disease protein DJ-1 is neuroprotective due to cysteine-sulfinic acid-driven mitochondrial localization. *Proc. Natl. Acad. Sci. U. S. A.* 101 9103–9108. 10.1073/pnas.0402959101 15181200 PMC428480

[B6] Chakrabarty P. Ceballos-Diaz C. Lin W.-L. Beccard A. Jansen-West K. McFarland N. R. (2011). Interferon-γ induces progressive nigrostriatal degeneration and basal ganglia calcification. *Nat. Neurosci.* 14 694–696. 10.1038/nn.2829 21572432 PMC3780582

[B7] Chang D. Nalls M. A. Hallgrímsdóttir I. B. Hunkapiller J. van der Brug M. Cai F. (2017). A meta-analysis of genome-wide association studies identifies 17 new Parkinson’s disease risk loci. *Nat. Genet.* 49 1511–1516. 10.1038/ng.3955 28892059 PMC5812477

[B8] Chen C. Turnbull D. M. Reeve A. K. (2019). Mitochondrial dysfunction in Parkinson’s disease-cause or consequence? *Biology* 8:38. 10.3390/biology8020038 31083583 PMC6627981

[B9] Costa J. Lunet N. Santos C. Santos J. Vaz-Carneiro A. (2010). Caffeine exposure and the risk of Parkinson’s disease: a systematic review and meta-analysis of observational studies. *J. Alzheimers. Dis.* 20(Suppl. 1) S221–S238. 10.3233/JAD-2010-091525 20182023

[B10] Dean C. Liu H. Dunning F. M. Chang P. Y. Jackson M. B. Chapman E. R. (2009). Synaptotagmin-IV modulates synaptic function and long-term potentiation by regulating BDNF release. *Nat. Neurosci.* 12 767–776. 10.1038/nn.2315 19448629 PMC2846764

[B11] Do C. B. Tung J. Y. Dorfman E. Kiefer A. K. Drabant E. M. Francke U. (2011). Web-based genome-wide association study identifies two novel loci and a substantial genetic component for Parkinson’s disease. *PLoS Genet.* 7:e1002141. 10.1371/journal.pgen.1002141 21738487 PMC3121750

[B12] Edgar R. Domrachev M. Lash A. E. (2002). Gene expression omnibus: NCBI gene expression and hybridization array data repository. *Nucleic Acids Res.* 30 207–210. 10.1093/nar/30.1.207 11752295 PMC99122

[B13] Fossati S. Giannoni P. Solesio M. E. Cocklin S. L. Cabrera E. Ghiso J. (2016). The carbonic anhydrase inhibitor methazolamide prevents amyloid beta-induced mitochondrial dysfunction and caspase activation protecting neuronal and glial cells in vitro and in the mouse brain. *Neurobiol. Dis.* 86 29–40. 10.1016/j.nbd.2015.11.006 26581638 PMC4713307

[B14] Kent W. J. Sugnet C. W. Furey T. S. Roskin K. M. Pringle T. H. Zahler A. M. (2002). The human genome browser at UCSC. *Genome Res.* 12 996–1006. 10.1101/gr.229102 12045153 PMC186604

[B15] Koga K. Mori A. Ohashi S. Kurihara N. Kitagawa H. Ishikawa M. (2006). 1H MRS identifies lactate rise in the striatum of MPTP-treated C57BL/6 mice. *Eur. J. Neurosci.* 23 1077–1081. 10.1111/j.1460-9568.2006.04610.x 16519673

[B16] Lee C. H. Della N. G. Chew C. E. Zack D. J. (1996). Rin, a neuron-specific and calmodulin-binding small G-protein, and Rit define a novel subfamily of ras proteins. *J. Neurosci.* 16 6784–6794. 8824319 10.1523/JNEUROSCI.16-21-06784.1996 PMC6579259

[B17] Lim N. Tesar S. Belmadani M. Poirier-Morency G. Mancarci B. O. Sicherman J. (2021). Curation of over 10 000 transcriptomic studies to enable data reuse. *Database* 2021:baab006. 10.1093/database/baab006 33599246 PMC7904053

[B18] Liscovitch N. French L. (2014). Differential co-expression between α-Synuclein and IFN-γ signaling genes across development and in Parkinson’s disease. *PLoS One* 9:e115029. 10.1371/journal.pone.0115029 25493648 PMC4262449

[B19] Lonsdale J. Thomas J. Salvatore M. Phillips R. Lo E. Shad S. (2013). The Genotype-Tissue Expression (GTEx) project. *Nat. Genet.* 45 580–585. 10.1038/ng.2653 23715323 PMC4010069

[B20] Lv Z. Krasnoslobodtsev A. V. Zhang Y. Ysselstein D. Rochet J. C. Blanchard S. C. (2016). Effect of acidic pH on the stability of α-synuclein dimers. *Biopolymers* 105 715–724. 10.1002/bip.22874 27177831 PMC4958566

[B21] Marras C. Beck J. C. Bower J. H. Roberts E. Ritz B. Ross G. W. (2018). Prevalence of Parkinson’s disease across North America. *NPJ Parkinsons Dis.* 4:21. 10.1038/s41531-018-0058-0 30003140 PMC6039505

[B22] Mendez J. A. Bourque M.-J. Fasano C. Kortleven C. Trudeau L.-E. (2011). Somatodendritic dopamine release requires synaptotagmin 4 and 7 and the participation of voltage-gated calcium channels. *J. Biol. Chem.* 286 23928–23937. 10.1074/jbc.M111.218032 21576241 PMC3129174

[B23] Millar T. Walker R. Arango J.-C. Ironside J. W. Harrison D. J. MacIntyre D. J. (2007). Tissue and organ donation for research in forensic pathology: the MRC sudden death brain and tissue bank. *J. Pathol.* 213 369–375. 10.1002/path.2247 17990279

[B24] Mistry M. Pavlidis P. (2010). A cross-laboratory comparison of expression profiling data from normal human postmortem brain. *Neuroscience* 167 384–395. 10.1016/j.neuroscience.2010.01.016 20138973 PMC2849877

[B25] Moriarty G. M. Olson M. P. Atieh T. B. Janowska M. K. Khare S. D. Baum J. (2017). A pH-dependent switch promotes β-synuclein fibril formation via glutamate residues. *J. Biol. Chem.* 292 16368–16379. 10.1074/jbc.M117.780528 28710275 PMC5625065

[B26] Nalls M. A. Blauwendraat C. Vallerga C. L. Heilbron K. Bandres-Ciga S. Chang D. (2019). Identification of novel risk loci, causal insights, and heritable risk for Parkinson’s disease: a meta-analysis of genome-wide association studies. *Lancet Neurol.* 18 1091–1102. 10.1016/S1474-4422(19)30320-5 31701892 PMC8422160

[B27] Navaroli D. M. Stevens Z. H. Uzelac Z. Gabriel L. King M. J. Lifshitz L. M. (2011). The plasma membrane-associated GTPase Rin interacts with the dopamine transporter and is required for protein kinase C-regulated dopamine transporter trafficking. *J. Neurosci.* 31 13758–13770. 10.1523/JNEUROSCI.2649-11.2011 21957239 PMC3205929

[B28] Nishimori I. Vullo D. Minakuchi T. Scozzafava A. Capasso C. Supuran C. T. (2013). Restoring catalytic activity to the human carbonic anhydrase (CA) related proteins VIII, X and XI affords isoforms with high catalytic efficiency and susceptibility to anion inhibition. *Bioorg. Med. Chem. Lett.* 23 256–260. 10.1016/j.bmcl.2012.10.103 23200251

[B29] Obergasteiger J. Castonguay A.-M. Frapporti G. Lobbestael E. Baekelandt V. Hicks A. A. (2021). RIT2 reduces LRRK2 kinase activity and protects against alpha-synuclein neuropathology. *bioRxiv* [Preprint]. 10.1101/2020.10.21.348144 PMC10042831 36973269

[B30] Pankratz N. Beecham G. W. DeStefano A. L. Dawson T. M. Doheny K. F. Factor S. A. (2012). Meta-analysis of Parkinson’s disease: identification of a novel locus, RIT2. *Ann. Neurol.* 71 370–384. 10.1002/ana.22687 22451204 PMC3354734

[B31] Payán-Gómez C. Riaño-Moreno J. Amador-Muñoz D. Ramírez-Clavijo S. (2019). Co-expression network analysis identifies possible hub genes in aging of the human prefrontal cortex. *Rev. Cienc. Salud* 17 201–222.

[B32] Pham C. L. L. Leong S. L. Ali F. E. Kenche V. B. Hill A. F. Gras S. L. (2009). Dopamine and the dopamine oxidation product 5,6-dihydroxylindole promote distinct on-pathway and off-pathway aggregation of α-synuclein in a pH-dependent manner. *J. Mol. Biol.* 387 771–785. 10.1016/j.jmb.2009.02.007 19361420

[B33] Piasecki E. (1999). Human acid-labile interferon alpha. *Arch. Immunol. Ther. Exp.* 47 89–98. 10202561

[B34] Pollard A. Shephard F. Freed J. Liddell S. Chakrabarti L. (2016). Mitochondrial proteomic profiling reveals increased carbonic anhydrase II in aging and neurodegeneration. *Aging* 8 2425–2436. 10.18632/aging.101064 27743511 PMC5115898

[B35] R Core (2013). *Team RA Language and Environment for Statistical Computing.* Vienna: R Core. 2013.

[B36] Ramasamy A. Trabzuni D. Guelfi S. Varghese V. Smith C. Walker R. (2014). Genetic variability in the regulation of gene expression in ten regions of the human brain. *Nat. Neurosci.* 17 1418–1428. 10.1038/nn.3801 25174004 PMC4208299

[B37] Rango M. Dossi G. Squarcina L. Bonifati C. (2020). Brain mitochondrial impairment in early-onset Parkinson’s disease with or without PINK1 mutation. *Mov. Disord.* 35 504–507. 10.1002/mds.27946 31898835

[B38] Scott L. Finley S. J. Watson C. Javan G. T. (2020). Life and death: a systematic comparison of antemortem and postmortem gene expression. *Gene* 731:144349. 10.1016/j.gene.2020.144349 31935499

[B39] Sinning A. Hübner C. A. (2013). Minireview: pH and synaptic transmission. *FEBS Lett.* 587 1923–1928. 10.1016/j.febslet.2013.04.045 23669358

[B40] Sjöblom B. Elleby B. Wallgren K. Jonsson B. H. Lindskog S. (1996). Two point mutations convert a catalytically inactive carbonic anhydrase-related protein (CARP) to an active enzyme. *FEBS Lett.* 398 322–325. 10.1016/s0014-5793(96)01263-x 8977131

[B41] Solesio M. E. Peixoto P. M. Debure L. Madamba S. M. de Leon M. J. Wisniewski T. (2018). Carbonic anhydrase inhibition selectively prevents amyloid β neurovascular mitochondrial toxicity. *Aging Cell* 17:e12787. 10.1111/acel.12787 29873184 PMC6052473

[B42] Sterky F. H. Trotter J. H. Lee S.-J. Recktenwald C. V. Du X. Zhou B. (2017). Carbonic anhydrase-related protein CA10 is an evolutionarily conserved pan-neurexin ligand. *Proc. Natl. Acad. Sci. U. S. A.* 114 E1253–E1262. 10.1073/pnas.1621321114 28154140 PMC5320979

[B43] Sun Y. Pham A. N. Hare D. J. Waite T. D. (2018). Kinetic modeling of pH-dependent oxidation of dopamine by iron and its relevance to Parkinson’s disease. *Front. Neurosci.* 12:859. 10.3389/fnins.2018.00859 30534046 PMC6275323

[B44] Tajima Y. (2010). Coffee-induced hypokalaemia. *Clin. Med. Insights Case Rep.* 3 9–13. 10.4137/ccrep.s4329 21769248 PMC3046007

[B45] Trabzuni D. Ryten M. Walker R. Smith C. Imran S. Ramasamy A. (2012). Quality control parameters on a large dataset of regionally dissected human control brains for whole genome expression studies. *J. Neurochem.* 120 473–473. 10.1111/j.1471-4159.2011.07602.x PMC3664422 21848658

[B46] Umek N. Geršak B. Vintar N. Šoštarič M. Mavri J. (2018). Dopamine autoxidation is controlled by acidic pH. *Front. Mol. Neurosci.* 11:467. 10.3389/fnmol.2018.00467 30618616 PMC6305604

[B47] Williams J. K. Yang X. Atieh T. B. Olson M. P. Khare S. D. Baum J. (2018). Multi-pronged interactions underlie inhibition of α-synuclein aggregation by β-synuclein. *J. Mol. Biol.* 430 2360–2371. 10.1016/j.jmb.2018.05.024 29782835 PMC6100766

[B48] Wingo T. Tu C. Laipis P. J. Silverman D. N. (2001). The catalytic properties of human carbonic anhydrase IX. *Biochem. Biophys. Res. Commun.* 288 666–669. 10.1006/bbrc.2001.5824 11676494

[B49] Zheng B. Liao Z. Locascio J. J. Lesniak K. A. Roderick S. S. Watt M. L. (2010). PGC-1α, a potential therapeutic target for early intervention in Parkinson’s disease. *Sci. Transl. Med.* 2 52ra73. 10.1126/scitranslmed.3001059 20926834 PMC3129986

[B50] Zhu Y. Wang L. Yin Y. Yang E. (2017). Systematic analysis of gene expression patterns associated with postmortem interval in human tissues. *Sci. Rep.* 7:5435. 10.1038/s41598-017-05882-0 28710439 PMC5511187

